# Comparison of molecular serotyping approaches of *Streptococcus agalactiae* from genomic sequences

**DOI:** 10.1186/s12864-017-3820-5

**Published:** 2017-06-01

**Authors:** Georgia Kapatai, Darshana Patel, Androulla Efstratiou, Victoria J. Chalker

**Affiliations:** 1 Respiratory and Vaccine Preventable Bacterial Reference Unit, Public Health England, London, UK; 2 Microbiology Reference Division, Public Health England, London, UK

**Keywords:** Group B Streptococci, Serotyping, Whole genome sequencing, *Streptococcus agalactiae*

## Abstract

**Background:**

Group B streptococcus (GBS) capsular polysaccharide is one of the major virulence factors underlying invasive GBS disease and a component of forthcoming vaccines. Serotype classification of GBS is based on the capsule polysaccharide of which ten variants are known to exist (Ia, Ib, II-IX). Current methods for GBS serotype assignment rely on latex agglutination or PCR while more recently a whole genome sequencing method was reported. In this study, three distinct algorithms for serotype assignment from genomic data were assessed using a panel of 790 clinical isolates.

**Methods:**

The first approach utilised the entire capsular locus coupled with a mapping methodology. The second approach continues from the first and utilised a SNP-based methodology across the conserved *cpsD-G* region to differentiate serotypes Ia-VII and IX. Finally the third approach used the variable *cpsG –K* region coupled with a mapping methodology. All three approaches were assessed for typeability (percentage of isolates assigned a serotype) and concordance to the latex agglutination methodology.

**Results:**

Following comparisons, the third approach using the variable *cpsG-K* region demonstrated the best performance with 99.9% typeability and 86.7% concordance. Overall, of the 105 discordant isolates, 71 were resolved following retesting of latex agglutination and whole genome sequencing, 20 failed to assign a serotype using latex agglutination and only 14 were found to be truly discordant on re-testing. Comparison of this final approach with the previously described assembly-based approach returned identical results.

**Conclusions:**

These results demonstrated that molecular capsular typing using whole genome sequencing and a mapping-based approach is a viable alternative to the traditional, latex agglutination-based serotyping method and can be implemented in a public health microbiology setting.

**Electronic supplementary material:**

The online version of this article (doi:10.1186/s12864-017-3820-5) contains supplementary material, which is available to authorized users.

## Background

*Streptococcus agalactiae* (group B streptococcus, GBS) is a leading cause of neonatal sepsis and meningitis worldwide [1]. Increasingly GBS is also an important cause of infections in immunosuppressed adults and the elderly [2]. A rise in the incidence of disease has been noted across multiple countries [3]. This is of particular concern because GBS is associated with a high morbidity and mortality [4].

Although no GBS vaccine is currently available conjugate polysaccharide vaccines covering the most common serotypes are in development [5]. Serotype classification of GBS is based on the capsule polysaccharide of which ten variants are known to exist (Ia, Ib, II-IX). The prevalence and distribution of serotypes differ between geographical regions, ethnic populations and clinical presentations [6]. The serotypes also differ in their virulence potential. Serotype III for example is associated with a significant proportion of neonatal disease particularly late-onset disease which presents between 7 to 89 days of age. In addition, serotype III is strongly associated with neonatal meningitis cases. The majority of serotype III isolates belong to multi-locus sequence type 17 which is associated with poor outcome of disease [7]. Accurate assignment of serotypes is important particularly for assessing serotype distributions in vaccine coverage and post-vaccine surveillance studies.

**Fig. 1:**
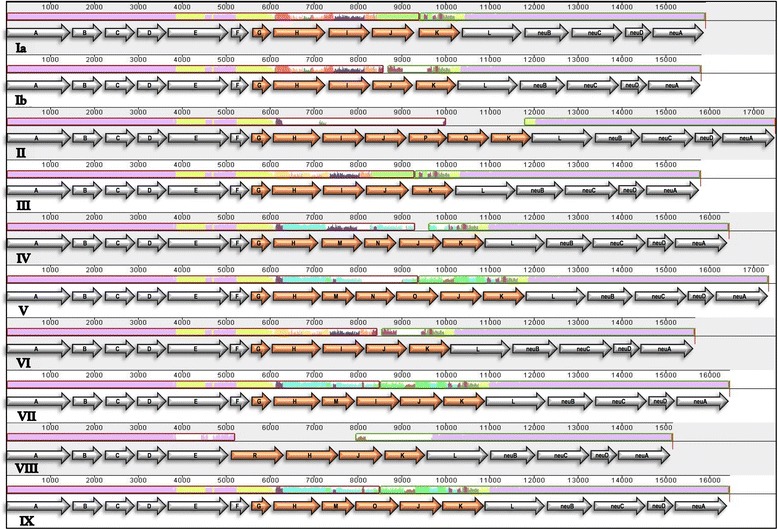
Comparison of the *cps* loci of all 10 serotypes (Ia, Ib, III-IX). The *cps* loci extracted from the reference strains where aligned using progressiveMauve and the gene regions were annotated using Artemis. The genes within the variable region *cpsG-K* are coloured in orange

The capsular polysaccharide is encoded on the *cps* locus and is composed of 16-18 genes [8]. The *cpsA* to -*F* genes are located at one end distal to the *cpsL*, *NeuB*, −*D*, −*A* and –*C* genes at the other, and these genes are highly conserved across the ten serotypes. In the central region from *cpsG* to –*K* in serotypes Ia-VII and IX and from *cpsR* to -*K* for serotype VIII the presence of genes and/or the sequence similarity varies between the serotypes (Fig. 1).

Multiple phenotypic serotyping methods such as latex agglutination, enzyme-based immunoassays and flow cytometry experiments using anti-capsular monoclonal antibodies have been described for GBS [9–11]. These assays can have limited typeability, can be subjective and are not able to assign all isolates to a type resulting in a high number of non-typeable isolates. Genotypic methods such as PCR-based DNA hybridisation, real-time PCR and restriction fragment length polymorphism assays can identify genetic variants in the *cps* locus that can be used to assign isolates to a serotype [12–15].

With the continuous reduction in cost of whole-genome sequencing (WGS) and the rapid development of bioinformatic infrastructures to analyse and store the large amount of data generated, WGS can provide a feasible approach to perform GBS serotyping. A recent study has described an approach to successfully determine the GBS serotype from WGS data [16]. The approach used was based on differences in the variable region of the *cps* locus and was able to assign isolates to all ten serotypes with high concordance with previous typing methods. In this study we sought to develop, compare and validate multiple WGS-based serotyping assays for GBS to determine the optimal methodology for implementation into a public health reference microbiology laboratory.

## Methods

### GBS reference strains and clinical isolates

A panel of 10 GBS reference strains (serotypes Ia to IX; Additional file 1: Table S1) were available from the archives of the Public Health England (PHE) Reference Laboratory.

The clinical isolates included in the study include isolates referred to the reference laboratory between April 2014 and December 2015 as well as retrospective isolates from 2010 for uncommon serotypes. In total, 790 isolates were used to compare and validate the WGS-based serotyping methods.

### Serotyping using latex agglutination

Isolates were cultured onto Columbia agar plates supplemented with horse blood (Oxoid Ltd., Hampshire, UK) and incubated aerobically at 37 °C for 24 h. Serological classification based on capsular polysaccharide types Ia, Ib and II to IX was performed using latex agglutination according to the manufacturer’s recommendations (Statens Serum Institute, Copenhagen, Denmark).

### DNA extraction and sequencing

Strains were pre-lysed in a lysis buffer composed of 2 mg lysozyme (Sigma-Aldrich, Dorset, UK), 120 U mutanolysin (Sigma-Aldrich), 400 μg RNase A (Qiagen, Manchester, UK), 20 μL proteinase K (>600 mAU/mL) and 15 μL of overnight culture. Cell suspensions were incubated for 1 h at 37 °C, 2 h at 56 °C followed by 1 h at 80 °C. DNA was extracted using the QIAsymphony SP system and the QIAsymphony DSP mini kit (Qiagen) according to the manufacturers’ recommendations. DNA concentrations were measured using the Quant-iT dsDNA Broad-Range Assay Kit (Life Technologies, Paisley, UK) and GloMaxR 96 Microplate Luminometer (Promega, Southampton, UK). For sequencing preparation, a Nextera XT DNA Library Preparation Kit (Illumina, San Diego, CA, USA) was used followed by sequencing using a HiSeq 2500 System (Illumina) and the 2 × 100-bp paired-end mode.

### Bioinformatic processing

Casava 1.8.2 (Illumina) was used to deplex the samples and FASTQ reads were processed with Trimmomatic to remove bases from the trailing end that fall below a PHRED score of 30 [17]. K-mer identification software (https://github.com/phe-bioinformatics/kmerid) was used to compare the sequence reads with a panel of curated NCBI Reference Sequence Database genomes to identify the species. FASTQ reads from all sequences in this study (*n* = 790) were submitted to the European Nucleotide Archive (ENA) using the ena_submission tool (https://github.com/phe-bioinformatics/ena_submission) and can be found at the PHE Pathogens BioProject PRJEB18093 at ENA (http://www.ebi.ac.uk/ena/data/view/PRJEB18093; Additional file 2: Table S2).

### Reference strain analysis

Ten GBS reference strains were sequenced and the genomic reads were assembled using SPAdes. The capsular locus sequences from all isolates were extracted using a BLAST query with the *cps* locus sequence of *Streptococcus agalactiae* strain A4 (accession number: DQ359707), a serotype V strain that covers the majority of the capsular genes [8, 18]. Artemis was used to annotate the capsular locus sequences [19]. Fastq reads (Accession numbers: ERS1462786 – ERS1462795) and annotated capsular locus sequences (Accession numbers: LT671983-LT671992; http://www.ebi.ac.uk/ena/data/view/LT671983-LT671992) for all reference strains are available in the PHE Pathogens BioProject PRJEB18093 at ENA (http://www.ebi.ac.uk/ena/data/view/PRJEB18093; Additional file 1: Table S1).

### De novo assembly

Genomic reads were assembled using SPAdes (version 2.5.1) de novo assembly software [20] with the following parameters ‘spades.py --careful -1 strain.1.fastq.gz -2 strain.2.fastq -t 4 -k 33,55,77,85,93’. The resulting contigs.fasta file was converted into a BLAST database using blast + (version 2.2.27) [21] and queried using selected query sequence.

### Sequence alignment

Capsular locus sequences were aligned using progressiveMauve [22] to visualise presence/absence of genes and define variable and conserved regions (Fig. 1). The *cps*D-G conserved regions were also aligned using MEGA6 [23] to investigate single nucleotide polymorphisms (SNP).

### Bioinformatic analysis for serotype prediction

Reads from each readset were mapped to a multi-fasta file containing the reference sequences using bowtie2 (version 2.1.0; following options used: --fr --minins 300 --maxins 1100 -k 99,999 -D 20 -R 3 -N 0 -L 20 -I S,1,0.50) [24]. A threshold coverage of >90% of the length of the sequence, minimum depth of 5 reads per bp and a mean depth of >20 reads over the entire length of the sequence was implemented during this step.

SAMtools (version 0.1.19) [25] mpileup was used to detect non-reference positions (SNPs) in each readset. BAQ computation was disabled and anonymous read pairs were included in variant calling (option –B and –A). The mpileup file was parsed and SNPs were filtered based on base quality (>30) and number of reads (>5).

## Results

### Selecting SNPs for serotype identification

Serotype identification using PCR amplification followed by Sanger sequencing was previously described by Kong et al. for serotypes Ia to VII and later by Slotvet et al. to include serotype IX [26, 27]. These two publications identified 52 single nucleotide polymorphisms (SNP) and one repeat region within the conserved *cpsD-G* region and based upon their results were able to differentiate serotypes Ia to VII and IX. Serotype VIII was not included due to an inability to amplify the target region using the primers detailed in those studies.

In this study, we examined the previously described SNPs using the capsular locus sequences from 10 reference strains. The capsular operon regions covering genes *cpsD* up to the 5′ end of *cpsG* for nine serotypes were aligned and all SNPs were recorded. Serotype VIII was excluded due to the lack of *cpsF* and *cpsG* genes; instead of the standard *cpsD-cpsE-cpsF-cpsG-cpsH* gene pattern, serotype VIII has *cpsD-cpsE-cpsR-cpsH* (Fig. 1)*.*

Previously published variants were compared to the results of the reference strain capsular locus alignment and 47 SNPs and the repeat region were concordant between the two sets. The SNPs at positions 138, 198, 249, 1173 and 1527 were not found in the respective reference strains (Additional file 3: Table S3). Furthermore, based on the alignment of the reference strains, 11 novel SNPs were identified that differentiated the nine serotypes. These were added to the 48 published variants and the new set of 59 variants was validated using clinical isolates.

Isolates were prospectively sequenced as referred from hospital laboratories therefore, at this initial stage of the study, genomic data were available for only a limited number of isolates. In total, 28 isolates were included in the initial validation in addition to the 10 reference strains; 3-6 isolates were selected for each serotype, except serotypes VI and IX where only one clinical isolate had been sequenced and serotype VII where no representative clinical isolates had been received during that period (Additional file 4: Table S4). Following analysis, serotype-specific SNPs were confirmed in clinical isolates for all serotypes except serotype III which as previously described is subdivided into different subtypes [26]. To simplify the process, all SNPs present only in certain serotype III subtypes were removed (*n* = 14), as well as the repeat region, leaving a final set of 44 SNPs (Table 1).

**Table 1:**
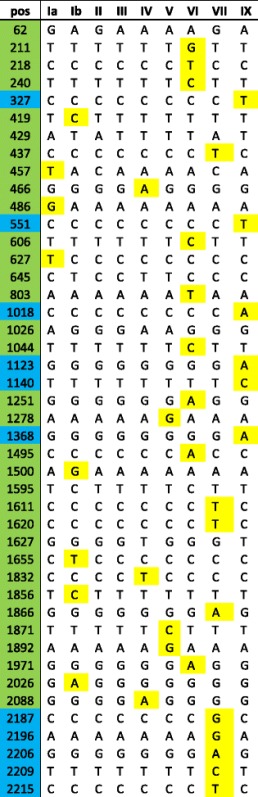
SNP sites used for identification of serotypes Ia-VII and XI in the conserved region, *cpsD* to 5' end of *cpsG*

Positions previously described [26, 27] are highlighted in green whereas SNPs identified in this study are highlighted in blue. SNPs highlighted in yellow are unique to a single serotype

### Assessment of targets for molecular capsular typing

#### Typeability

Firstly, the entire capsular locus was used as a target; reads were mapped to the capsular loci extracted from the 10 reference strains and serotype was assigned based on mapping coverage along the length of the reference sequence (mapping coverage >90%). Using this approach, a single serotype was assigned in 163 cases (20.6%) whereas in one case the highest percentage mapping coverage observed was below 90% resulting in a ‘Failed’ tag and in the remaining 629 cases the method was not able to distinguish between two serotypes that both exhibited >90% coverage. This result demonstrated higher than 90% similarity between the capsular sequences of serotypes Ia and III (*n* = 535), IX and V (*n* = 71) and IX and VII (*n* = 17) (Additional file 5: Table S5). This was confirmed by aligning the two sequences within each pair using BLAST. The high similarity between Ia and III was also previously described by Chaffin et al. [28]. Contamination was suspected for the remaining four cases, each corresponding to different pairs, considering that the percent coverage seen using BLAST was <90%. The 167 cases where a serotype was assigned successfully, correspond mainly to five serotypes (Ib, *n* = 51; II, *n* = 68; IV, *n* = 29; VI, *n* = 10; VIII, *n* = 2), with the remaining two cases corresponding to samples assigned Ia and IX, respectively (Table 2). The serotypes corresponding to the respective pairs (Ia/III and IX/VII) were missed due to mapping coverage slightly below 90% (88.11% and 89.78%, respectively).

**Table 2:** Detailed summary of the results (typeability and concordance) of the three WGS methods

		cpsG-K			Full CPS			SNP-based				Grant total
		TRUE	FALSE	NT	TRUE	FALSE	NT	TRUE	FALSE	NT	n/a^a^	
Latex agglutination	Ia	155	16			4	167	151	15	5		179
	Ib	44	16		43	9	8	43	14	3		61
	II	47			47			41		6		48
	III	336	18			6	348	320	16	18		378
	IV	20	1		20		1	20	1			22
	IX	9	4		1	2	10	9	3	1		13
	V	57	10			7	60	55	9	3		70
	VI	7	3	1	7		4	7	3	1		11
	VII	7					7	7				7
	VIII	2	8		2	2	6		8		2	10
	NT		29			12	17		27	2		30
	Grant total	684	105	1	120	42	628	653	96	39	2	829

TRUE corresponds to concordant with latex agglutination results, FALSE corresponds to discordant, NT is non-typeable whereas n/a is not applicable for the method (relevant only for the SNP approach)

^**a**^Serotype VIII with both latex agglutination and Full CPS

Secondly, SNPs within the conserved *cpsD-G* region were investigated amongst serotypes Ia-VII and IX. The reads were first mapped to the full capsule and any isolates with serotype VIII capsule were precluded from further analysis (*n* = 2). Reads were mapped to the *cpsD-G* region of *S. agalactiae* strain 3139 (region 3166-5955: accession number AF332908.1) and a serotype was assigned based on the bases present in the 44 SNP positions described above (Table 1). Using this approach, serotype was assigned to 749 cases (94.8%) whereas in the remaining 39 cases, serotype was not assigned due to either low base mapping (*n* = 17), a mixed base (variant present in 20-80% of the reads) in certain SNP positions (*n* = 2) or an incomplete SNP profile match (*n* = 20) (Additional file 6: Table S6). In two cases no SNPs were detected; one of the isolates had also failed with the previous method with a highest percent coverage of 71.93% whereas the second isolate was the Ia isolate called as a single serotype by the previous method. Further investigation, revealed that the failed isolate, when mapped to the full capsular locus reference file, had no reads mapping in the region corresponding to the 3’end of *cpsC – cpsH* inclusive. Similar investigation for the Ia isolate revealed a smaller region of no coverage within the *cpsD-G* region (1549-2069 bps), which covered 12 of the 44 SNP positions. Due to the low coverage (<90%) the SNP analysis was not performed. However, based on the 32 SNP positions that were covered this isolate matched (32/32) the serotype Ia SNP profile.

The variable region *cpsG-K* (Fig. 1) was the third and final region to be evaluated in this study. This region was previously successfully used to assign serotype from WGS data using de novo assembly followed by BLAST analysis [16]. In this study, a mapping approach was used with the *cpsG-K* regions extracted from the capsular locus sequences of the 10 reference strains used as a reference. This approach assigned a serotype to 789 of the isolates (99.9%) and the one isolate that failed with highest coverage of 66.97% was the same isolate that failed with the two previous methods.

#### Concordance

Concordance of the three molecular serotyping assays was observed compared to the latex agglutination method. Concordance was observed in 120 of the 162 typed isolates (74.1%) using the full capsular locus approach (Table 3). The 42 discordant isolates were investigated by repeat serotyping and/or WGS. In 22 cases, discordances were resolved; in seven cases repeat WGS was concordant with the serotype whereas in the remaining 15 cases repeating the serotype analysis gave a result concordant with the WGS. The one failed isolate mentioned previously was also investigated; originally typed serologically as serotype VI, repeat serological analysis was not able to assign a serotype due to auto-agglutination with all sera.

**Table 3:** Investigation of discordant isolates in this dataset (*n* = 107)

SampleID	cpsG-K	Full CPS	SNP-based	SNPhits	Original Results				Retested		
					LAB_SEROTYPE	G-KvsLAB	CPSvsLAB	SNPvsLAB	Retested lab	Retested WGS	Resolved
PHEGBS0002	II	II	Serotype undetermined	*n* = 42/44	IX	FALSE	FALSE	NT	NT		No
PHEGBS0007	Ia	Ia,III	Ia	*n* = 44/44	IX	FALSE	NT	FALSE	Ia		Yes
PHEGBS0016	II	II	II	*n* = 44/44	IX	FALSE	FALSE	FALSE	Ib	II	No
PHEGBS0017	V	V,IX	V	*n* = 44/44	VI	FALSE	NT	FALSE	NT		No
PHEGBS0022	V	V,IX	V	*n* = 44/44	VIII	FALSE	NT	FALSE	NT		No
PHEGBS0024	Ia	Ia,III	Ia	*n* = 44/44	VIII	FALSE	NT	FALSE	Ia		Yes
PHEGBS0025	Ia	Ia,III	Ia	*n* = 44/44	IX	FALSE	NT	FALSE	Ia		Yes
PHEGBS0026	Ia	Ia,III	Ia	*n* = 44/44	VIII	FALSE	NT	FALSE	Ia		Yes
PHEGBS0027	Ia	Ia,III	Ia	*n* = 44/44	VIII	FALSE	NT	FALSE	Ia		Yes
PHEGBS0028	II	II	II	*n* = 44/44	VIII	FALSE	FALSE	FALSE	Ia	II	No
PHEGBS0029	Ib	Ib	Ib	*n* = 44/44	VIII	FALSE	FALSE	FALSE	Ib		Yes
PHEGBS0030	V	V,IX	V	*n* = 44/44	VIII	FALSE	NT	FALSE	NT		No
PHEGBS0033	V	V,IX	V	*n* = 44/44	VI	FALSE	NT	FALSE	NT		No
PHEGBS0035	Ia	Ia	Serotype undetermined	*n* = 0/44	NT	FALSE	FALSE	NT	Ia		Yes
PHEGBS0037	VI	VI	VI	*n* = 44/44	III	FALSE	FALSE	FALSE	VI		Yes
PHEGBS0043	Ia	Ia,III	Ia	*n* = 44/44	NT	FALSE	NT	FALSE	Ia		Yes
PHEGBS0070	Ia	Ia,III	Ia	*n* = 44/44	NT	FALSE	NT	FALSE	Ia		Yes
PHEGBS0075	II	II	II	*n* = 44/44	NT	FALSE	FALSE	FALSE	II		Yes
PHEGBS0082	V	V,IX	V	*n* = 44/44	NT	FALSE	NT	FALSE	V		Yes
PHEGBS0084	IV	IV	IV	*n* = 44/44	Ib	FALSE	FALSE	FALSE	IV		Yes
PHEGBS0086	Ia	Ia,III	Ia	*n* = 44/44	NT	FALSE	NT	FALSE	Ia		Yes
PHEGBS0091	II	II	II	*n* = 44/44	NT	FALSE	FALSE	FALSE	Ia	II	No
PHEGBS0098	V	V,IX	V	*n* = 44/44	NT	FALSE	NT	FALSE	V		Yes
PHEGBS0103	IV	IV	IV	*n* = 44/44	Ib	FALSE	FALSE	FALSE	IV		Yes
PHEGBS0134	Ia	Ia,III	Ia	*n* = 44/44	IV	FALSE	NT	FALSE	Ia		Yes
PHEGBS0152	Ia	Ia,III	Ia	*n* = 44/44	NT	FALSE	NT	FALSE	Ia		Yes
PHEGBS0157	IV	IV,III	IV	*n* = 44/44	Ib	FALSE	NT	FALSE	IV		Yes
PHEGBS0163	II	II	Serotype undetermined	*n* = 42/44	Ib	FALSE	FALSE	NT	Ia	II	No
PHEGBS0167	III	III,Ia	III	*n* = 44/44	NT	FALSE	NT	FALSE	III		Yes
PHEGBS0176	III	III,Ia	Serotype undetermined	*n* = 42/44	V	FALSE	NT	NT	NT		No
PHEGBS0195	Ia	Ia,III	Ia	*n* = 44/44	III	FALSE	NT	FALSE	III	III	Yes
PHEGBS0196	Ia	Ia,III	Ia	*n* = 44/44	Ib	FALSE	NT	FALSE	Ib	Ib	Yes
PHEGBS0198	Ib	Ib	Ib	*n* = 44/44	V	FALSE	FALSE	FALSE	V	V	Yes
PHEGBS0200	V	V,IX	V	*n* = 44/44	Ia	FALSE	NT	FALSE	Ia	Ia	Yes
PHEGBS0201	III	III,Ia	III	*n* = 44/44	Ia	FALSE	NT	FALSE	Ia	Ia	Yes
PHEGBS0203	Ia	Ia,III	Ia	*n* = 44/44	V	FALSE	NT	FALSE	V	V	Yes
PHEGBS0204	Ia	Ia,III	Ia	*n* = 44/44	III	FALSE	NT	FALSE	III	III	Yes
PHEGBS0205	V	V,IX	V	*n* = 44/44	III	FALSE	NT	FALSE	III	III	Yes
PHEGBS0208	IV	IV	IV	*n* = 44/44	V	FALSE	FALSE	FALSE	V	V	Yes
PHEGBS0209	III	III,Ia	III	*n* = 44/44	Ia	FALSE	NT	FALSE	Ia	Ia	Yes
PHEGBS0210	V	V,IX	V	*n* = 44/44	Ia	FALSE	NT	FALSE	Ia	Ia	Yes
PHEGBS0211	Ia	Ia,III	Ia	*n* = 44/44	III	FALSE	NT	FALSE	III	III	Yes
PHEGBS0212	Ia	Ia,III	Ia	*n* = 44/44	III	FALSE	NT	FALSE	III	III	Yes
PHEGBS0215	III	III,Ia	III	*n* = 44/44	Ia	FALSE	NT	FALSE	Ia	Ia	Yes
PHEGBS0216	III	III,Ia	Serotype undetermined	*n* = 42/44	Ib	FALSE	NT	NT	Ib	Ib	Yes
PHEGBS0217	Ia	Ia,III	Ia	*n* = 44/44	III	FALSE	NT	FALSE	III	III	Yes
PHEGBS0218	Ib	Ib	Ib	*n* = 44/44	III	FALSE	FALSE	FALSE	III	III	Yes
PHEGBS0220	III	III,Ia	III	*n* = 44/44	Ib	FALSE	NT	FALSE	Ib	Ib	Yes
PHEGBS0222	Ib	Ib	Ib	*n* = 44/44	III	FALSE	FALSE	FALSE	III	III	Yes
PHEGBS0225	III	III,Ia	III	*n* = 44/44	Ia	FALSE	NT	FALSE	Ia	Ia	Yes
PHEGBS0227	Ia	Ia,III	Ia	*n* = 44/44	Ib	FALSE	NT	FALSE	Ib	Ib	Yes
PHEGBS0228	III	III,Ia	III	*n* = 44/44	Ia	FALSE	NT	FALSE	Ia	Ia	Yes
PHEGBS0229	Ib	Ib	Ib	*n* = 44/44	V	FALSE	FALSE	FALSE	V	V	Yes
PHEGBS0231	V	V,IX	V	*n* = 44/44	III	FALSE	NT	FALSE	III	III	Yes
PHEGBS0232	Ia	Ia,III	Ia	*n* = 44/44	Ib	FALSE	NT	FALSE	Ia		Yes
PHEGBS0233	IV	IV	IV	*n* = 44/44	III	FALSE	FALSE	FALSE	III	III	Yes
PHEGBS0236	III	III,Ia	III	*n* = 44/44	Ib	FALSE	NT	FALSE	Ib	Ib	Yes
PHEGBS0238	Ib	Ib	Ib	*n* = 44/44	III	FALSE	FALSE	FALSE	III	III	Yes
PHEGBS0240	III	III,Ia	III	*n* = 44/44	Ia	FALSE	NT	FALSE	Ia	Ia	Yes
PHEGBS0241	III	III,Ia	III	*n* = 44/44	VI	FALSE	NT	FALSE	VI	VI	Yes
PHEGBS0242	Ia	Ia,III	Ia	*n* = 44/44	III	FALSE	NT	FALSE	III	III	Yes
PHEGBS0264	II	II	II	*n* = 44/44	NT	FALSE	FALSE	FALSE	NT	II	No
PHEGBS0266	IV	IV	IV	*n* = 44/44	Ib	FALSE	FALSE	FALSE	NT	IV	No
PHEGBS0271	II	II	II	*n* = 44/44	NT	FALSE	FALSE	FALSE	II		Yes
PHEGBS0313	IV	IV	IV	*n* = 44/44	Ib	FALSE	FALSE	FALSE	IV		Yes
PHEGBS0327	IV	IV	IV	*n* = 44/44	Ib	FALSE	FALSE	FALSE	IV		Yes
PHEGBS0352	II	II	II	*n* = 44/44	Ib	FALSE	FALSE	FALSE	II		Yes
PHEGBS0373	V	V,IX	V	*n* = 44/44	NT	FALSE	NT	FALSE	NT	V	No
PHEGBS0390	II	II	II	*n* = 44/44	V	FALSE	FALSE	FALSE	V	II	No
PHEGBS0394	II	II	II	*n* = 44/44	NT	FALSE	FALSE	FALSE	II		Yes
PHEGBS0398	II	II	II	*n* = 44/44	NT	FALSE	FALSE	FALSE	V	II	No
PHEGBS0404	II	II	II	*n* = 44/44	NT	FALSE	FALSE	FALSE	NT	II	No
PHEGBS0432	II	II	II	*n* = 44/44	V	FALSE	FALSE	FALSE	NT		No
PHEGBS0433	IV	IV	IV	*n* = 44/44	Ib	FALSE	FALSE	FALSE	IV		Yes
PHEGBS0460	Ia	Ia,III	Ia	*n* = 44/44	III	FALSE	NT	FALSE	NT		No
PHEGBS0492	III	III,Ia	III	*n* = 44/44	NT	FALSE	NT	FALSE	III		Yes
PHEGBS0520	V	V,IX	V	*n* = 44/44	NT	FALSE	NT	FALSE	NT		No
PHEGBS0554	II	II	II	*n* = 44/44	NT	FALSE	FALSE	FALSE	NT		No
PHEGBS0568	II	II	II	*n* = 44/44	Ia	FALSE	FALSE	FALSE	NT		No
PHEGBS0586	II	II	II	*n* = 44/44	V	FALSE	FALSE	FALSE	NT		No
PHEGBS0600	II	II	Serotype undetermined	*n* = 41/44	III	FALSE	FALSE	NT	NT		No
PHEGBS0603	III	III,Ia	III	*n* = 44/44	NT	FALSE	NT	FALSE	NT		No
PHEGBS0607	VII	VII,IX	VII	*n* = 44/44	III	FALSE	NT	FALSE	III		No
PHEGBS0610	III	III,Ia	III	*n* = 44/44	Ia	FALSE	NT	FALSE	Ia		No
PHEGBS0611	Ia	Ia,III	Ia	*n* = 44/44	III	FALSE	NT	FALSE	III		No
PHEGBS0615	Ia	Ia,III	Ia	*n* = 44/44	V	FALSE	NT	FALSE	Ia		Yes
PHEGBS0620	Ia	Ia,III	Serotype undetermined	*n* = 41/44	III	FALSE	NT	NT	III	III	Yes
PHEGBS0627	II	II	II	*n* = 44/44	Ib	FALSE	FALSE	FALSE	Ia		No
PHEGBS0640	III	III,Ia	III	*n* = 44/44	VIII	FALSE	NT	FALSE	III		Yes
PHEGBS0641	III	III,Ia	III	*n* = 44/44	NT	FALSE	NT	FALSE	III		Yes
PHEGBS0642	III	III,Ia	III	*n* = 44/44	Ia	FALSE	NT	FALSE	III		Yes
PHEGBS0643	III	III,Ia	III	*n* = 44/44	Ia	FALSE	NT	FALSE	Ia		No
PHEGBS0644	III	III,Ia	III	*n* = 44/44	NT	FALSE	NT	FALSE	III		Yes
PHEGBS0671	IV	IV	Serotype undetermined	*n* = 38/44	Ia	FALSE	FALSE	NT	Ia	IV	No
PHEGBS0728	II	II	II	*n* = 44/44	NT	FALSE	FALSE	FALSE	NT		No
PHEGBS0737	II	II	II	*n* = 44/44	V	FALSE	FALSE	FALSE	V		No
PHEGBS0738	V	V,IX	Serotype undetermined	*n* = 42/44	Ia	FALSE	NT	NT	V		Yes
PHEGBS0761	V	V,IX	V	*n* = 44/44	NT	FALSE	NT	FALSE	V		Yes
PHEGBS0772	Ia	Ia,III	Serotype undetermined	*n* = 38/44	NT	FALSE	NT	NT	Ia		Yes
PHEGBS0821	VI	VI	VI	*n* = 44/44	NT	FALSE	FALSE	FALSE	VI		Yes
PHEGBS0822	Ib	Ib	Ib	*n* = 44/44	NT	FALSE	FALSE	FALSE	Ib		Yes
PHEGBS0823	Ia	Ia,III	III	*n* = 44/44	NT	FALSE	NT	FALSE	NT		No
PHEGBS0824	Ib	Ib	Ib	*n* = 44/44	Ia	FALSE	FALSE	FALSE	Ia	Ib	No
PHEGBS0825	VII	VII,IX	VII	*n* = 44/44	NT	FALSE	NT	FALSE	VII		Yes
PHEGBS0826	VI	VI	VI	*n* = 44/44	Ia	FALSE	FALSE	FALSE	VI		Yes

The SNP-based approach resulted in concordance with serology for 653 of 749 typed isolates (87.2%). The remaining 96 discordant isolates were investigated by repeating serology and/or WGS. Discordance was resolved in 67 cases; in 29 cases repeat WGS was concordant with the laboratory serotype whereas in the remaining 37 cases repeating the serological analysis gave a result concordant with the WGS. Of the 96 discordant isolates, 37 were also discordant with the previous method; the remaining five were non-typeable by the SNP-based method (Table 3).

Finally, using the variable *cpsG-K* region concordance was observed in 684 of the 789 cases (86.7%). The remaining 105 discordant isolates, included all discordant isolates from the first method (*n* = 42) and 95/96 of the discordant isolates from the second method, in addition to 10 non-typeable by the SNP-based method. All discordant isolates were retested with latex agglutination by an investigator blinded to the previous results. One isolate discordant with the SNP-based method was correctly assigned serotype Ia with the *cpsG-K* method; using the full capsule method the serotype pair Ia/III was called and with the SNP-based method serotype III; following retesting in the laboratory serotype V was assigned which did not agree with either method. Further investigation into this isolate, using mapping analysis and de novo assembly followed by BLAST analysis, revealed that whereas the *cpsD-G* region corresponded to serotype III (44/44 SNP matches) this isolate contained a serotype Ia *cpsH* gene [28], suggesting a recombination event may have taken place. The serological method was repeated for all discordant isolates (*n* = 105) and in 40 cases the new result corresponded to the predicted serotype (by WGS) whereas in 20 cases no serotype was assigned due to lack of reaction or reaction with multiple sera. Of the remaining 45 discordant cases, 44 were re-sequenced and in 31 of these cases the new predicted serotype was concordant with the serological result. Overall following retesting only 14 cases remain truly discordant excluding the 20 non-typeable by sera (Table 3).

#### Comparison of mapping and assembly approach

The results of the third mapping-approach, that uses the variable *cpsG-K* region, were compared to the previously described assembly method followed by BLAST [16]. All 790 isolates were assembled and serotype was assigned following BLAST analysis of the contigs against the variable *cpsG-K* region from all 10 serotypes. The results of this approach were concordant with the results from the mapping approach for 721/790 isolates (Additional file 2: Table S2). One isolate that had previously failed with the mapping approach, with all three methods, due to low coverage of the capsular operon region (depth > = 5 is used as threshold) return no hits with BLAST. Serotype was not assigned for the remaining 68 isolates due to lack of a complete match; the *cpsG-K* region was present but split into two or more contigs. Further investigation in a subset of these cases (*n* = 20) revealed that in all cases the serotype assigned by mapping was also the serotype with the highest coverage along the multiple contigs in the assembly/BLAST approach.

## Discussion

The conserved region *cpsD-G* was previously used for molecular capsular typing in Group B streptococci using a SNP-based approach with PCR amplification and Sanger sequencing [26, 27], whereas more recently the variable region *cpsG-K* was used to predict serotype from WGS data using a de novo assembly and BLAST analysis approach [16]. In this study, these two capsular locus target regions as well as the full capsular locus sequence were investigated for their efficacy in predicting serotype in GBS from WGS data. We used a mapping approach followed by SNP-based approach for the *cpsD-G* region on a validation panel of 790 GBS isolates.

The full capsular locus approach was able to predict 5/10 serotypes (Ib, II, IV, VI, VIII) with distinct capsular locus sequences but was unable to distinguish the remaining five, including serotype III which is the most prevalent serotype in the UK. These latter serotypes were resolved to one of two highly similar capsular loci (Ia/III, V/IX, VII/IX) each sharing more than 90% similarity. Previous publications have reported the similarity of the serotype Ia and III capsular loci; the main difference lies in the *cpsH* gene [28]. Further investigation into the three serotypes (V, VII & IX) of the remaining two pairs (V/IX & VII/IX) showed high sequence variability for *cpsM* based on the read mapping patterns for serotypes V and IX; that is in addition to the presence of *cpsN* in serotype V (V: *cpsG-cpsH-cpsM-cpsN-cpsO-cpsJ-cpsK,* IX: *cpsG-cpsH-cpsM-cpsO-cpsJ-cpsK*). For the VII/IX pair, the two serotypes differ in the presence of *cpsO* in serotype IX and *cpsI* in VII (VII: *cpsG-cpsH-cpsM-cpsI-cpsJ-cpsK*). Furthermore, fragmented mapping (reads mapping to some region but not others) between *cpsM* and *cpsK* suggests high sequence variability in *cpsJ* as well.

The SNP-based approach failed to predict a serotype in 39 cases due to low mapping coverage or a mixed base at certain SNP positions for approximately half the cases; this dependence on specific positions is one of the disadvantages of using a SNP-based methodology coupled with mapping and strict quality criteria (depth > = 5 and base quality > = 30). However, the remaining unassigned cases were of particular concern; in these cases failure to predict serotype was due to a mixed SNP profile, this is a 41-43/44 match to a serotype-specific SNP profile with one to three SNP positions matching the expected base of another serotype. The positions affected include 12 SNPs located in *cpsE* and *cpsF*, including 10 previously published (419, 429, 437, 457, 486, 645, 1026, 1611, 1627 and 1866). However, based on previous studies [26], three of these positions have alternative variants in serotype III subtypes. Specifically, at position 437, the presence of a T instead of a C is the only difference between serotype II and III-4 SNP profiles of region *cpsD-G*; three isolates have 43/44 SNPs matching the serotype II SNP profile and a T in position 437 suggesting that these isolates are indeed serotype III subtype 4 which also agrees with the serological result and the result from the *cpsG-K* method. Furthermore, in positions 486 and 1026 the presence of G and A instead of A and G, respectively are indicative of serotype III subtype 3 and based on this, three isolates with a partial 42/44 serotype III profile and G and A in 486 and 1026 respectively would be assigned serotype III subtype 3. This also agrees with the cpsG-K assignment and in one of the cases with the serological result. For the remaining 7 positions, the presence of these alternate variants, even though occurring rarely, suggests that variants at these positions are not conserved within a serotype.

Finally, mapping the reads to the variable *cpsG-K* region produced the highest typeability (99.9%) with only a single isolate remaining non-typeable. However, this isolate was non-typeable with all methods and was shown to lack a large part of the capsular locus (*cpsC-H)* therefore the lack of serotype prediction was correct. Using this method 86.7% concordance was observed with only 105 isolates, which included all (but one) of the discordant isolates from the two previous methods and 10 non-typeable isolates by the SNP method. Therefore, this number represents the discordance between the molecular and serological approach. Following retesting using both serological and WGS methods only 14 isolates remained truly discordant.

The initially high discordance rate can mainly be attributed to the traditional methodology of latex agglutination which can be subjective; specifically re-serotyping using latex agglutination resulted in the same serotype in only 39/105 cases indicating latex agglutination or lab transcription error. This high ambiguity with the serological method may be in part due to a problem with a particular batch as some of the sera used were shown to agglutinate poorly when tested with controls. This included the Ia sera resulting in a number of serotype Ia isolates (*n* = 18) being mis-identified as rarer types or non-typeables and was resolved when a new batch was used. Furthermore, isolates referred to the reference lab are inoculated onto an agar plate and following overnight incubation a sweep of material is usually tested for serotyping whereas for retesting subculture from a single colony was used. Therefore the presence of a mixed population in the initial culture could result in ambiguous serotyping results downstream in the investigation. However not all discordance can be attributed to the traditional methodology. Cases where the traditional re-serotyping was consistent but repeat WGS resulted in a different serotype could be attributed to a laboratory transcription error.

Overall, this study demonstrated that molecular capsular typing using WGS is a viable alternative to the traditional serotyping method and the most efficient method between the three investigated was using a mapping approach on the variable *cpsG-K* method. These results further support the evidence provided by Sheppard et al. [16], however, unlike the assembly-based methodology used in that study, a mapping approach as described here is more appropriate for use in reference microbiology. Generally, mapping is faster and can provide more precise, region-specific, quality metrics than genome assembly. Furthermore, comparisons of the two approaches, revealed lower typeability with the assembly approach followed by blasting (721/790 vs 789/790 with the mapping approach) due to incomplete assembly of the capsular operon region.

## Conclusions

The use of genomics to determine serotype of GBS is critical to Public Health, improving on current subjective methodologies, enabling determination of serotype in strains that were previously designated nontypeable using phenotypic testing. Ultimately it will enable testing, remote determination of serotype and collation of data from strains without reliance of specialised testing centres. The ability to determine the serotype is essential to enable identification of cross infections linked to healthcare associated infection and monitoring of specific serotypes in differing patient groups. It will have increasing importance when considering the serological profile of the bacterial population in response to forthcoming vaccinations.

## Additional files

Additional file 1: Table S1. Information on historical GBS strains used as reference for the 10 serotypes (Ia-IX). (XLSX 11 kb)

Additional file 2: Table S2. Metadata for all clinical isolates used in this study (*n* = 790). (XLSX 36 kb)

Additional file 3: Table S3. Investigation of the previously published SNPs using the 10 reference strains. SNPs highlighted in yellow do not correlate with the respective reference and are removed from subsequent analysis. (XLSX 19 kb)

Additional file 4: Table S4. Investigation of the SNPs (previously published and novel) identified using multiple alignment of the reference *cps* loci using a small a small dataset of clinical samples (*n* = 28). SNP positions highlighted in grey represent SNPs not found in all isolates of a particular serotype. These positions were removed and the remaining SNPs represent the final set used in this study. (XLSX 23 kb)

Additional file 5: Table S5. Sequence analysis for the serotype pairs identified using the full *cps* locus approach. Hit coverage corresponds to the mapping coverage observed during analysis whereas the BLAST coverage corresponds to the percent coverage seen during pairwise alignment using BLAST. (XLSX 8 kb)

Additional file 6: Table S6. Investigation of the not-typeable (“Serotype undetermined”) isolates from the SNP-based approach. The pileup file created during the analysis was investigated in detail to determine the aetiology behind the incomplete SNP profiles. (XLSX 19 kb)

## Abbreviations

ENA: European Nucleotide Archive
GBS: Group B streptococcus
PCR: Polymerase chain reaction
PHE: Public Health England
SNP: Single nucleotide polymorphism
WGS: Whole genome sequencing
